# Protective effects of madecassoside against Doxorubicin induced nephrotoxicity in vivo and in vitro

**DOI:** 10.1038/srep18314

**Published:** 2015-12-14

**Authors:** Zhonghao Su, Jin Ye, Zhenxia Qin, Xianting Ding

**Affiliations:** 1 School of Biomedical Engineering, Institute for Personalized Medicine, Shanghai Jiao Tong University, Shanghai 200030, China; 2 School of Basic Medicine, Shanghai University of Traditional Chinese Medicine, Shanghai 201203, China

## Abstract

Madecassoside (MA), a triterpenoid saponin isolated from C. asitica, exerts various pharmacological activity including antioxidative and antinflammatory. Doxorubicin (DOX), a common chemotherapeutic drug, has been reported to induce numerous toxic side effects including renal-toxicity. We hypothesized that MA administration may decrease renal-toxicity caused by DOX. In this study, we investigated this hypothesis by introducing MA and DOX into the culture of Human Proximal Tubule Cells HK-2 and mice model. Our *in vivo* study demonstrated that MA (12 mg/kg), treatment for two weeks attenuated DOX-induced renal injury via protecting renal function, recovering antioxidant enzyme activity, inhibiting Bax, p-ERK1/2, NF-κB p65, iNOS expression and increasing Bcl-2 expression. Similar findings were obtained in our *in vitro* studies with treatment of DOX and/or MA. Further studies with application of iNOS inhibitor and ERK1/2 kinase inhibitor indicated that the inhibitory effects of MA on DOX-induced apoptosis and inflammation might be mediated by the suppression of the activation of cleaved caspase-3, ERK1/2 pathways, NF-κB p65 and NO production. These results suggest that MA is a promising protective agent for DOX-induced renal toxicity and can be a potential candidate to protect against renal toxicity in DOX-treated cancer patients.

Centella asiatica (Umbelliferae), growing in tropical swampy areas, is an annual herbaceous plant used for treating renal disease in traditional Chinese medicine. Centella asiatica has been reported to contain a large number of compounds belonging to different pharmacological classes. Triterpene compounds, isolated from Centella asiatica, are believed to be the most biologically active and medicinal values ingredients^1^. Madecassoside (MA, Fig. 1), among the Triterpene compounds, is one of the major triterpene glycosides^2^. MA was found to be effective in decreasing oxidative stress and enhancing the activities of antioxidative enzymes in various disease models^3^,^4^,^5^,^6^. It was reported that MA can inhibit lipopolysaccharide-induced cardiac dysfunction in rat and TNF-α production in rat cardiomyocytes^3^, reduce ischemia-reperfusion injury in rat^4^, attenuate inflammatory response in mice with collagen-induced arthritis^5^ and facilitate burn wound healing in mice^6^.

As an important antitumor anthracycline (ANT) antibiotic, Doxorubicin(DOX) is commonly used to treat a variety of malignant neoplasms, including breast cancer, leukemia^7^ and solid tumors^8^. However, due to its severe side effects, such as cardiotoxicity^7^, nephrotoxicity^9^ and hepotoxicity^10^, DOX has been carefully reconsidered as chemotherapy medicine. It is now recognized that the Multi-organ injury of DOX is partially due to its oxidative damage^11^,^12^. DOX has highly potent cytotoxicity to induce Human Proximal Tubule Cells HK-2 cells apoptosis by significantly changing caspase activities^13^. Although MA has shown anti-inflammatory and anti-oxidant effects in multiple disease models, whether it could inhibit DOX-induced cell apoptosis has not been examined.

Herein, we investigated the hypothesis that the presence of MA reduces DOX-induced nephrotoxicity. We introduced MA and DOX into mice and Human Proximal Tubule Cells HK-2 cells. In our *in vivo* study, we evaluated renal function, antioxidant enzymes activity and analyzed histopathological features. Furthermore, we studied the underlying molecular mechanisms. Multiple standard assays were used to examine the cell toxicity, including 3-(4,5-dimethylthiazol-2-yl)-2,5-diphenyltetrazolium bromide (MTT), Terminal deoxynucleotidyl transferase dUTP nick end labeling (tunel), flow cytometry and western blot analysis. Our results indicate that MA administration can significantly attenuate DOX-induced HK-2 cell apoptosis. DOX altered the expression of Bcl-2, Bax, Caspase-3, Caspase-9, APAF1, the phosphorylated-extracellular-signal regulated kinases 1 and 2(p-ERK1/2), nuclear factor-kappaB (NF-κB). The addition of MA could recover these aberrant protein expressions back to normal basal level. Our results indicate that the inhibitory effects of MA on DOX-induced apoptosis and inflammation might be mediated by the suppression of the activation of cleaved caspase-3, ERK1/2 pathways, NF-κB p65 and Nitric oxide (NO) production. Our results support the application of MA as a protective agent in chemotherapy medicine optimization and practice.

## Results

### *In Vivo* Studies

#### Effect of Treatment on Mortality and Body Weight

**Figure 2:**
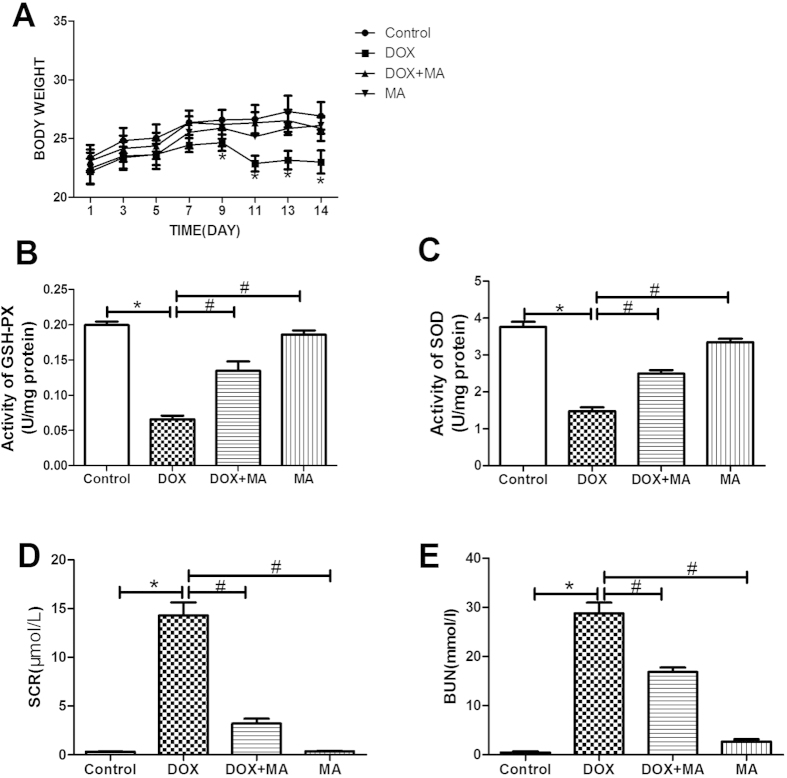
Measurement of mice body weight, renal functions and antioxidant enzymes changes after DOX and/or MA treatment. Abbreviations: DOX: doxorubicin; SOD: superoxide dismutase; GSH-PX: glutathione peroxidase; BUN: Blood Urea Nitrogen; SCR: Serum Creatinine. (**A**) DOX treatment caused severe reduction of body weight compared to control group. (**B**,**C**) DOX + MA treatment decreased antioxidant enzymes in the kidney SOD and GSH-PX content respectively compared to DOX group. (**D**,**E**) DOX + MA treatment protected renal function by reducing the level of SCR and BUN compared to DOX group. (n = 9). Animals were treated for 2 weeks. *P < 0.05 vs. control; ^#^*P* < 0.05 vs. DOX.

36 mice were randomly distributed into 4 groups: Control group, MA-only group, DOX-only group and MA + DOX group. After two week treatments, the mice were sacrificed. No mortality was observed in control, DOX + MA and MA goups. However, DOX group showed 10% mortality (1 out of 10 mice) (Data was not shown). Furthermore, after one week treatment, DOX treatment led to severe reduction of body weight compared to non-treatment control group, while the addition of MA rescued the DOX induced body loss (Fig. 2A).

#### Effect of Treatment on Antioxidant Enzymes

Since MA has antioxidative function, we wondered if MA could inhibit the effect of DOX on antioxidants enzymatic (SOD, GSH-PX) in mice kidney. Administration of DOX led to reduction of SOD and GSH-PX content compared to control group respectively in the kidney. On the contrary, concomitant administration of MA with DOX restored enzyme activities towards that of the control group (Fig. 2B,C).

#### Effect of Treatment on Kidney Functions

Serum BUN and creatinine(SCR) are indicators of kidney function. Our data demonstrated that Bun and SCR were significantly increased in DOX group compared to control group. MA administration alone did not exhibit any significant change versus the control group. MA + DOX showed reduced BUN and SCR levels in serum compared to DOX group (Fig. 2D,E).

#### Histological Evaluation

**Figure 3:**
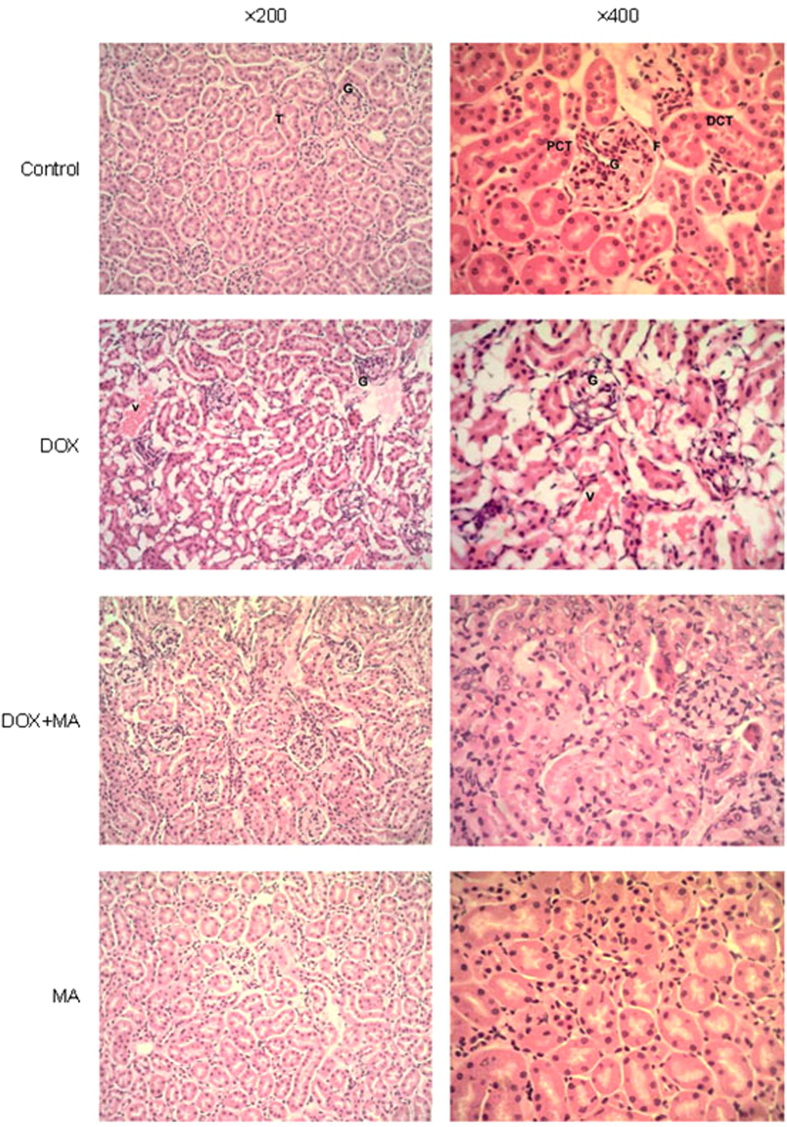
Kidney section prepared from tissues of different treatment groups and stained by HE staining. G: renal glomerulus T: renal tubules, F: filtration space, PCT: proximal convoluted tubules, DCT: distal convoluted tubule, V: vascul. Control and MA group showed normal pathology. DOX treated group showed renal glomerulus gross distortion, renal tubules atrophy necrosis and exfoliated and vascular congestion. However, DOX + MA combination group showed little pathology changes.

Histopathological examination with HE staining revealed that control and MA groups showed normal renal glomeruli and cortical tubules structures. However, DOX-treated group displayed glomeruli distortion, filtration space obliterated disappear, tubules focal atrophy necrosis and exfoliation, and vascular congestion. DOX + MA combination group showed little pathology changes, confirming the bioavailability of MA *in vivo* (Fig. 3).

#### Effect of MA on apoptosis and inflammation related proteins in mice kidney

**Figure 4:**
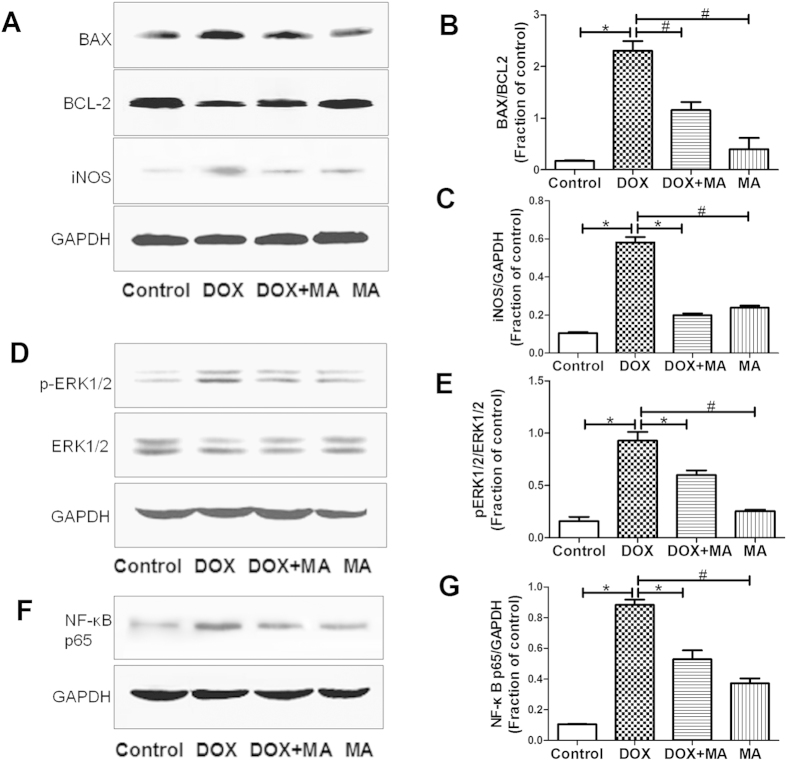
Apoptosis and inflammation related protein expressions were determined by Western blot analysis in DOX and/or MA-treated mice kidney. (**A,B**) The ratio of Bax/Bcl-2 was increased significantly in DOX Group versus Control Group, which was recovered in DOX + MA Group. (**D,E**) The increased level of p-ERK1/2 was shown in DOX Group versus Control Group, which was attenuated in DOX + MA Group. (**F,G**) Total NF-κB p65 expression was increased in DOX Group versus Control Group, which was relieved in DOX + MA Group. (**A–C**) Expression of iNOS was increased in DOX Group versus Control Group, which was blocked in DOX + MA Group. Each bar represent mean ± SEM from groups of 5–9 mice. *P < 0.05 vs. control; ^#^*P* < 0.05 vs. DOX.

To investigate DOX induced apoptosis and inflammation, we probed for the related proteins such as Bax, Bcl-2, phosphorylated-ERK, NF-κB p65 and iNOS by western blot analysis. Our data showed that the ratio of Bax/Bcl-2, p-ERK, NF-κB p65 and iNOS were increased in DOX group. These changes were attenuated significantly in DOX + MA Group (Fig. 4).

### *In Vitro* Studies

#### Doxorubicin-induced apoptosis in HK-2 cells

**Figure 5:**
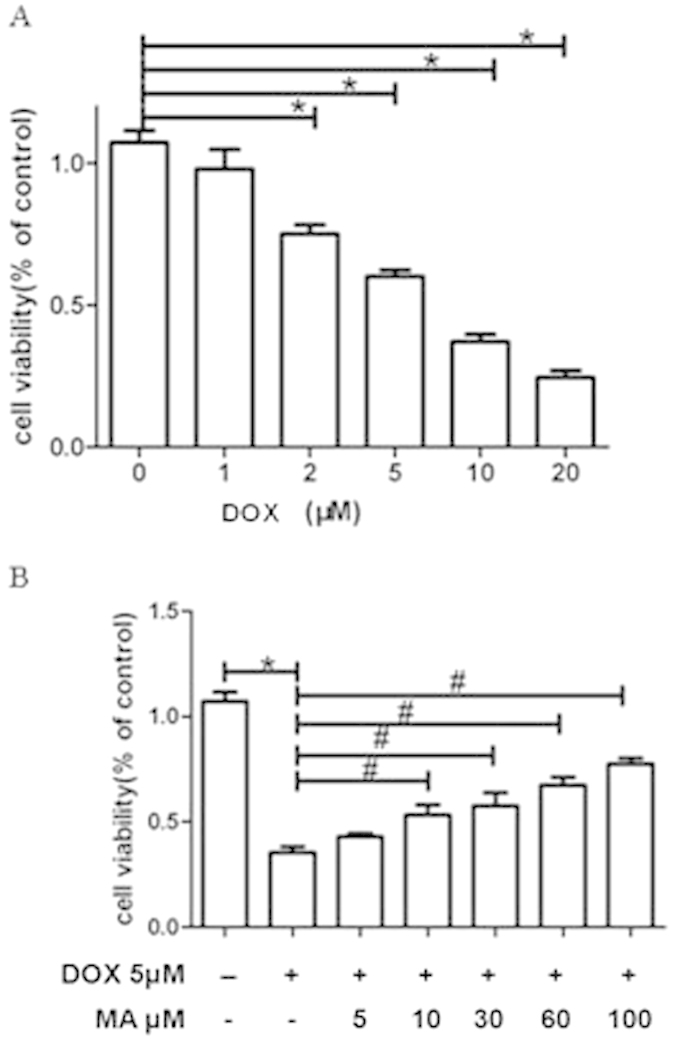
MA reduced DOX toxicity by promoting HK-2 cell viability. (**A**) DOX treatment led to severe reduction of cell viability. (**B**) With the presence of MA, DOX induced toxicity was relieved and the HK-2 cell metabolic activity was recovered as indicated by the MTT assays. *P < 0.05 vs. control; ^#^*P* < 0.05 vs. DOX.

We measured DOX-induced cytotoxicity in HK-2 cells. Cells were treated with various DOX concentrations (0–20 μM). The viability of cells were determined using MTT assay. A significant reduction in cell survival was observed after the treatment with DOX. The cytotoxic effect became obvious as the DOX dose increased, with an IC50 dose of approximately 5 μM (Fig. 5A).

#### Madecassoside prevented Doxorubicin-induced apoptosis in HK-2 cells

In order to investigate the protective effect of MA on DOX-induced cell damage, MA dosed from 5 μM to 100 μM were introduced into DOX treated HK-2 cells. After incubation with MA for 24 h, cell viability was determined using the MTT assay as described in the method section. Notably, an increase in cell viability of approximately 40% could be detected in response to 10–100 μM of MA compared to the group that was only treated with DOX (Fig. 5B).

#### Madecassoside prevented Doxorubicin -induced nitric oxide production in HK-2 cells

**Figure 6:**
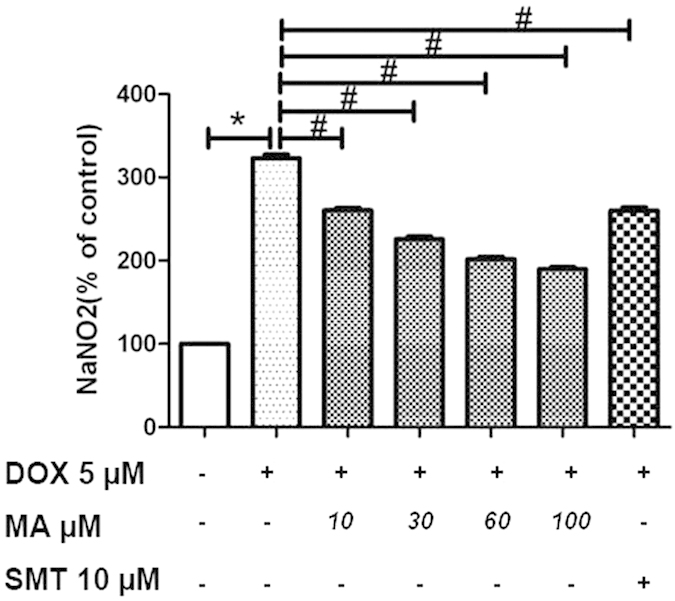
Nitric oxide (NO) secretion was determined after Doxorubicin or/and MA treatment in HK-2 cells. HK-2 cells were treated with 5 μM of DOX and different dose of MA for 24 h. The amount of NO was calculated using a standard curve. DOX induced extra NaNO_2_ production, while the addition of MA or SMT(S-methylisothiourea hemisulfate) could obviously reduce DOX induced NaNO_2_ secretion. Experiments were independently performed three times. *P < 0.05 vs. control; ^#^*P* < 0.05 vs. DOX.

NO plays an important role in oxidative stress and inflammation. iNOS, inducible nitric oxide synthase, is an enzyme in the body to produce NO. In order to study the role of NO production in DOX-induced apoptosis and inflammation, an iNOS inhibitor SMT (S-methylisothiourea hemisulfate) was applied to inhibit NO production. After incubation SMT with DOX at concentrations of 5 μM for 24 h, the amount of NO was calculated using a standard curve. NO can cause renal toxicity by reacting with superoxide radicals to form peroxynitrite, a cytotoxic agent that induces peroxidation of lipid membranes. Our data showed that NaNO_2_ was increased after DOX incubation, which was attenuated after SMT administration. Interestingly, DOX-increased NaNO_2_ production was also reduced with MA incubation in a dose-dependent manner (Fig. 6).

#### Madecassoside attenuated Doxorubicin -induced apoptotic in HK-2 cells

**Figure 7:**
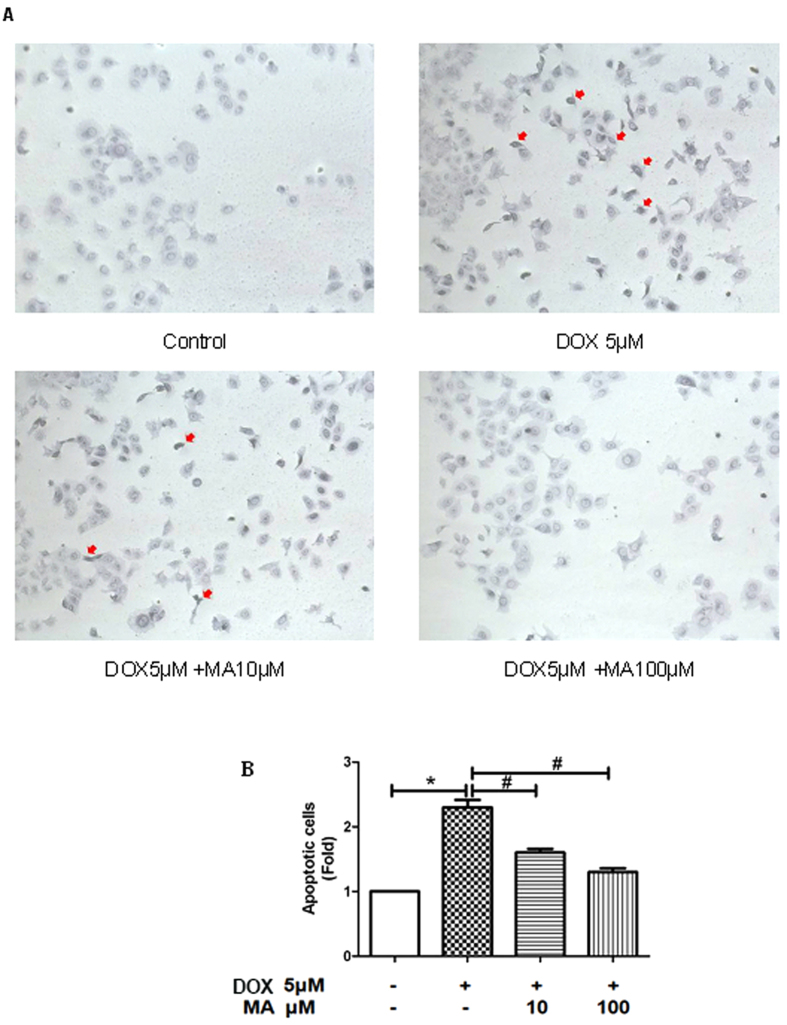
MA reduces DOX-induced apoptosis. (**A**) Tunel stains (apoptotic cells stain Purple-blue) shows representative apoptosis of HK-2 cells after 24 h exposure to DOX. (**B**) The number of cells with apoptotic bodies was counted in 5 randomly chosen fields at 400 × magnification and the percentage of apoptosis was calculated as the average count of these 5 fields. Results are presented as means ± SEM of 3 individual experiments. *P < 0.05 vs. control; ^#^*P* < 0.05 vs. Dox.

To confirm the protective effects of MA on DOX-induced HK-2 cells toxicity as shown in Fig. 5B, we further examined whether DOX could induce cell apoptosis and MA could repress it by both microscopy and flow cytometry. Apoptotic cells were examined by tunel assay. The apoptotic cells were observed as purple-blue in DOX-treated HK-2 cells (Fig. 7A, red arrow). When the cells were co-treated with DOX and MA, the addition of 10 μM MA attenuated the apoptosis and the addition of 100 μM MA almost reduced apoptosis percentage close to that of the control group (Fig. 7B).

**Figure 8:**
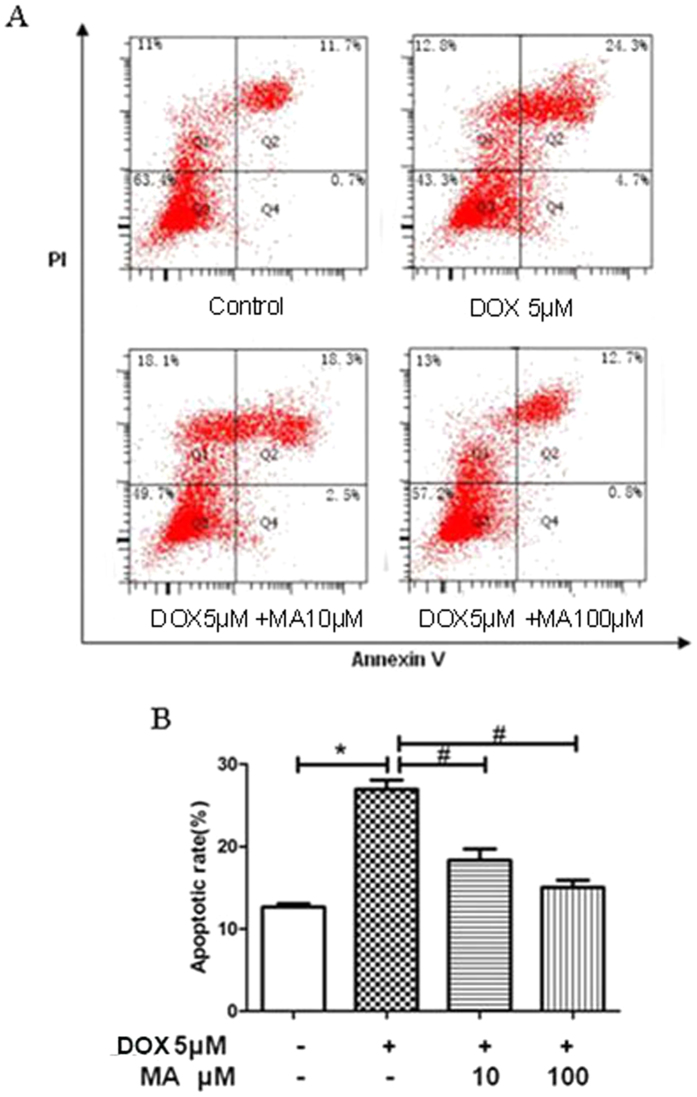
Flow cytometric analysis of HK-2 cells following DOX and MA stimulation *in vitro*. (**A**) flow cytometry for HK-2 cells stained with annexin V (apoptosis) and propidium iodide (PI; necrosis) following 24 h stimulation of MA at varied concentrations (10,100 μM). (**B**) Quantification of annexin V-positive cells (Q2 + Q4) was performed using an annexin V/PI kit. Percentage of HK-2 cell apoptosis (flow cytometry) after incubation DOX and/or MA was plotted.

Furthremore, Annexin-V and PI staining were applied to confirm apoptotic changes in DOX-treated HK-2 cells. Double stained cells were analyzed by flow cytometry. The results revealed that HK-2 cells treated with DOX for 24 h exhibited late apoptosis in annexin-V (+)/PI (+) staining (apoptotic cells), which was prevented by MA treatment (Fig. 8).

#### Effects of Madecassoside on apoptosis and inflammation related proteins in HK-2 cells

**Figure 9:**
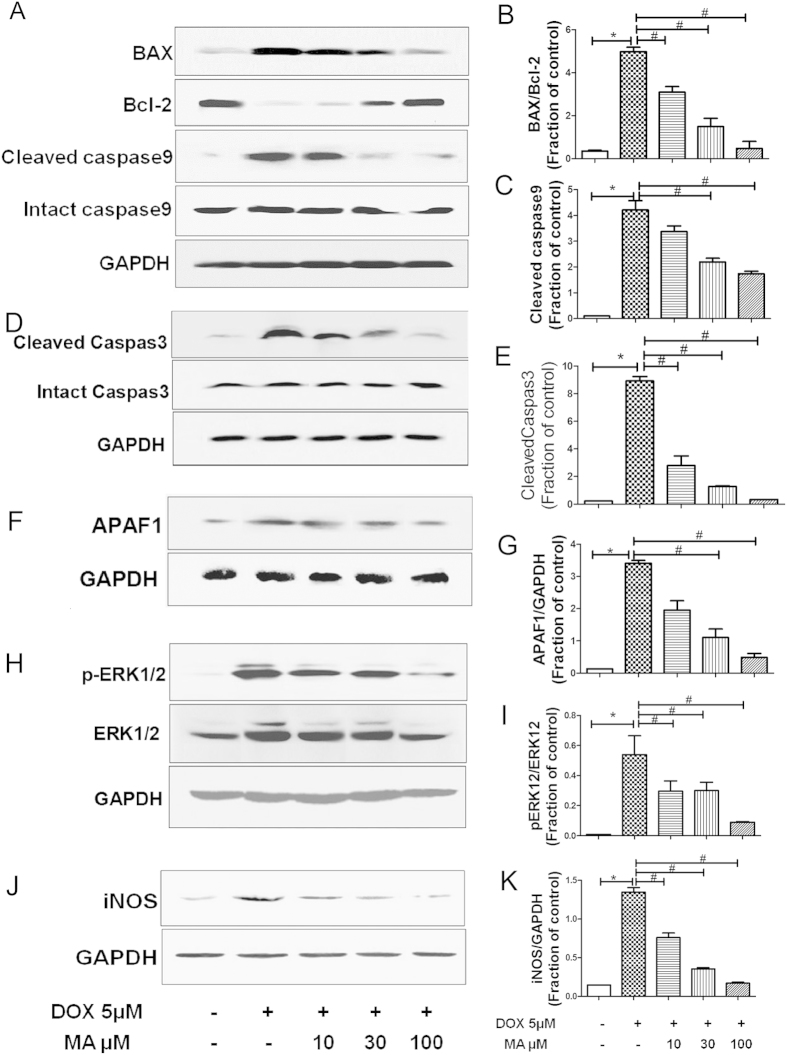
Western Blot analysis for apoptosis and inflammation related proteins in HK-2 cells. (**A–K**) Effect of MA on apoptosis and inflammation related proteins in HK-2 cells incubated with DOX. (**A,B**) Western blot indicated that the level of proapoptotic marker Bax increased in DOX-treated HK-2 cells, whereas that of anti-apoptotic protein Bcl-2 decreased. (**A–C**) Increased cleaved caspase-9, (**D,E**) cleaved caspase-3, (**F,G**) APAF1, (**H,I**) p-ERK1/2, (**J,K**) iNOS expressions were observed in DOX treated HK-2 cells. MA combined with DOX reduced these changes. Results are presented as means ± SEM of 3 individual experiments. *P < 0.05 vs. control; ^#^*P* < 0.05 vs. DOX.

To study the protective effect of MA on DOX-induced apoptosis and inflammation related proteins expressions of Bax, Bcl-2(Fig. 9A,B), intact and cleaved caspase-9(Fig. 9A–C), caspase-3(Fig. 9D,E), Apaf-1(Fig. 9F,G), p-ERK1/2(Fig. 9H,I) and iNOS (Fig. 9J,K) were measured by western blot analysis. It was found that ratio of Bax/Bcl-2, cleaved caspase-9, caspase-3, Apaf-1, p-ERK1/2, iNOS were increased after DOX incubation. MA combined with DOX reduced these changes (Fig. 9).

#### Effects of Madecassoside on activation of NF-κB p65 in HK-2 cells

**Figure 10:**
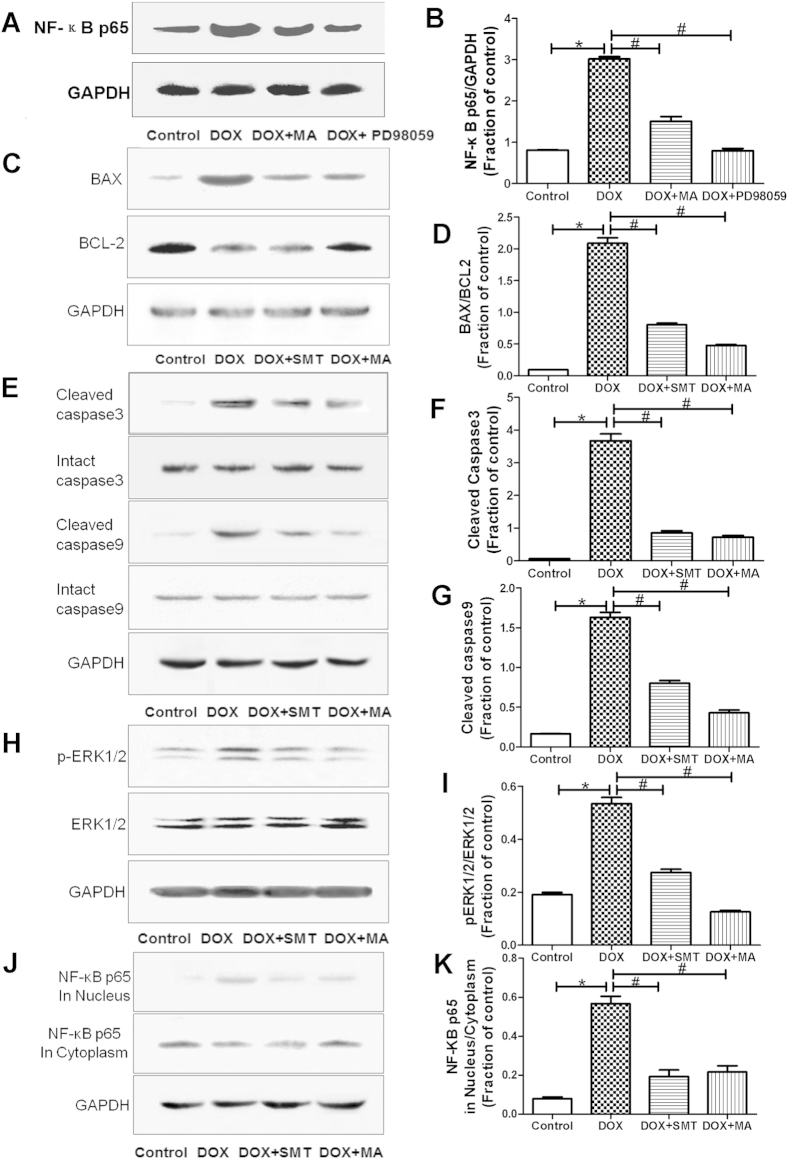
Detection of apoptosis-related protein expressions, activation of ERK1/2 and NF-κB p65 in HK-2 cells. (**A,B**) Pretreatment with MA (30 μM) for 1 h before exposure to 5 μM DOX for 24 h suppressed the over expression of nuclear NF-κB p65. Incubation with PD98059 (ERK inhibitor, 10 μM) for 1 h before exposure to DOX attenuated the expression of the nuclear NF-κB p65 subunit. (**C–G**) HK-2 cells were exposed to 5 μM DOX for 24 h after pretreatment with MA (30 μM) or SMT, an iNOS inhibitor, (10 μM) for 1 h. Effect of MA and SMT on DOX-induced apoptosis-related proteins including Bax, Bcl-2, caspase-3 and caspase-9 in HK-2 cells. (**H,I**) Effect of MA and SMT on DOX-induced p-ERK1/2. (**J,K**) Effect of MA and SMT on DOX-induced activation of NF-κB p65, both in Nucleus and Cytoplasm. Results are presented as means ± SEM of 3 individual experiments. *P < 0.05 vs. control; ^#^*P* < 0.05 vs. DOX.

NF-κB p65 can be activated by p-ERK1/2, moreover, the p-ERK1/2 and increased NF-κB p65 in mice kidney were observed after DOX injection in our studies. Therefore, p-ERK1/2 and NF-κB p65 expression was further investigated after incubation with DOX and/or MA in HK-2 cells. Similarly, DOX incubation induced p-ERK1/2 and NF-κB p65 expression, which could be inhibited by co-incubation with MA. Meanwhile, the expression of total ERK1/2 remained unaffected. To detect the role of ERK1/2 in DOX-induced HK-2 cells apoptosis, we treated HK-2 cells with PD98059 (ERK inhibitor, 10 μM, Selleck, USA) for 1 h before DOX (5 μM) treatment. As shown in Fig. 10A,B, the expression of NF-κB p65 was affected by DOX for 24 h compared with untreated controls, while the pretreatment with PD98059 could suppress the increased expression of nuclear NF-κB p65. Likewise, MA pretreatment for 1 h also attenuated DOX-induced expression of NF-κB p65. These findings suggest that MA might play a protective role in DOX-induced apoptosis through ERK-mediated NF-κB p65 activation (Fig. 10A,B).

#### Effects of Madecassoside on NO-involved signaling pathway in Doxorubicin-induced HK-2 cells apoptosis

To further investigate the mechanism of MA protective effect on DOX-induced HK-2 cells apoptosis, SMT, an iNOS inhibitor was used to alter NO production. As shown in Fig. 10, application of SMT decreased DOX-induced apoptosis-related proteins including the ratio of Bax/Bcl-2, activated caspase-3 and caspase-9 (Fig. 10C–G). Moreover, SMT reduced DOX-activated ERK1/2 and NF-κB p65 by decreasing the p-ERK1/2 and the translocation of NF-κB p65 to nucleus from cytoplasm (Fig. 10H–K). Interestingly, MA exhibited similar effect on DOX-induced apoptosis in HK-2 cells in the same manner. All these findings indicate that MA might inhibit DOX-induced apoptosis in renal disease through NO-involved signaling pathway like its role in focal cerebral ischemia reperfusion injury^14^ (Fig. 10C–K).

## Discussion

Nephrotoxicity is one of the most common adverse effects of DOX as an anti-cancer drugs^15^. DOX is known to interact with DNA by intercalation, causing inhibition of macromolecular biosynthesis, and to inhibit the role of the enzyme topoisomerase II during transcription and replication^16^. Although the exact mechanism of DOX-induced nephrotoxicity remains unknown, it is believed to be mediated through free radical formation, oxidative damage, and membrane lipid peroxidation. Madecassoside (MA) plays an important role in attenuating oxidative stress and inflammatory response^17^,^18^. The major findings of the present study are that MA can prevent apoptosis and inflammatory responses induced by DOX through the suppression of the activation of cleaved caspase-3, ERK1/2 pathways, NF-κB p65 and NO production.

In our *in vivo* study, we found that DOX was harmful to kidney, which was mainly reflected by (1) decreasing the body weight; (2) impairing renal function; (3)reducing antioxidant enzyme activity such as SOD and GSH-PX; (4) formation of distorted morphology structure in kidney. Moreover, all these alteration could be inhibited by MA. In order to investigate the molecular mechanism of DOX-induced nephrotoxicity, we measured the expressions of protein BAX, Bcl-2, p-ERK1/2, NF-κB p65 and iNOS. Our results suggested DOX caused nephrotoxicity by affecting the expressions of these proteins, which are involved in apoptosis and inflammation. Meanwhile, MA was found to reduce DOX-induced changes of these protein expressions.

DOX can cause apoptosis by triggering the mitochondrial pathway through the activation of caspase-3 in renal proximal tubular cells^19^. In order to explore the possible relationship in details, we carried out further studies in HK-2 cells. Mitochondria-dependent apoptosis is characterized by formation of a multi-protein complex called an apoptosome consisting of Apaf-1, Cyto-c and Cleaved caspase-9 after their release from mitochondria^20^. In our study, DOX treatment increased apoptotic molecules expressions, such as cleaved caspase-3, cleaved caspase-9, Apaf-1 and the ratio of Bax/Bcl-2. All these changes were attenuated by MA, suggesting MA can inhibit DOX-induced mitochondrial-dependent apoptosis in HK-2 cells through the suppressions of cleaved caspase-3 expression, apoptosome formation and the ratio of Bax/Bcl-2.

Dysregulation of inflammation and apoptosis is involved in DOX-caused renal injury. ERK1/2 and NF-κB p65 are involved in the signaling pathway of inflammation and apoptosis. It is reported that DOX could induce NF-κB and ERK1/2 activation both *in vivo* and *in vitro*^21^,^22^,^23^. However, little evidence is currently available on the relationship between DOX induced cell apoptosis and NF-κB and/or ERK1/2 activation. We examined the involvement of ERK1/2/NF-κB p65 signaling molecules that have been implicated as stimulators of inflammation and apoptosis. In our study, we observed that DOX-induced NF-κB p65 activation was prevented both by MA and ERK inhibitor. It was demonstrated MA pretreatment in DOX-treated HK-2 cells decrease expression of NF-κB p65. Our results indicate that MA inhibit the expression of NF-κB p65 probably through ERK1/2 mediated pathways.

NO, as an important inflammatory molecule, plays a dual role in acute renal injury. iNOS, inducible nitric oxide synthase, is an enzyme in the body to produce NO. NO increasing represents DOX-mediated oxidative stress in HK-2 Cells^24^. During the early stages, a small amount of NO produced by vascular endothelial cells can protect against renal injury through its vasodilator, anti-platelet, and anti-inflammatory activities. Conversely, excessive NO can cause renal toxicity by reacting with superoxide radicals to form peroxynitrite, a cytotoxic agent that induces peroxidation of lipid membranes^25^. In order to study the role of NO production in DOX-induced apoptosis and inflammation, iNOS inhibitor SMT was applied to inhibit NO production. Our results showed that SMT inhibited DOX-induced expression of apoptosis-related protein Bax, Bcl-2, caspase-3 and caspase-9, and activation of NF-κB p65 and ERK1/2. More significantly, MA was also found to effectively reduce DOX induced NO levels. In addition, MA could prevent DOX-decreased antioxidant enzyme activity such as SOD and GSH-PX. These results indicate that MA is a potential superoxide radical scavenging agent that protects cells from DOX induced oxidative stress.

**Figure 11:**
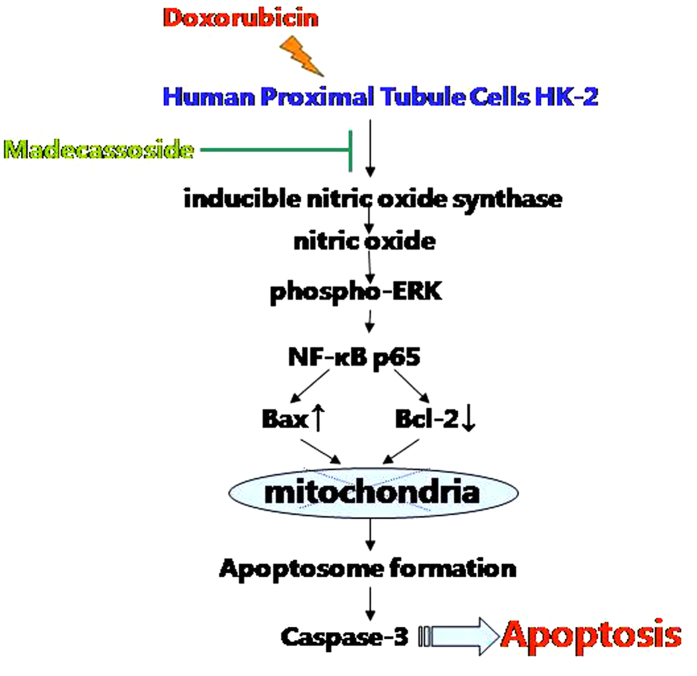
Possible signaling pathways involved in the protective effects of Madecassoside against Doxorubicin induced HK-2 cell apoptosis.

In summary, MA inhibits DOX-stimulated HK-2 cells apoptosis in a concentration-dependent manner. The inhibitory effects of MA on DOX-induced apoptosis and inflammation might be mediated by the suppression of the activation of cleaved caspase-3 and ERK1/2 pathways, NF-κB p65 and NO production as shown in Fig. 11. Therefore, our results suggest that MA may serve as a valuable protective agent in renal toxicity and inflammatory diseases. The results presented indicate that MA could be a beneficial compound for the clinical application for chemotherapy patient.

## Methods

### Materials

MA (C_48_H_78_O_20_, MW:975.12, purity: ≥98%) determined by HPLC as previously described (Gunther and Wagner,1996) was purchased from from China National Medicines Co. Ltd. (Shanghai, China). Dimethyl sulfoxide (DMSO), and MTT were obtained from Sigma Chemical Co. (St. Louis, MO, USA). Rabbit anti-Bcl-2, Bax, Caspase-3, Caspase-9, APAF1, p-ERK1/2, NF-κB p65 and GAPDH were purchased from Cell Signaling Technology (Danvers, MA, USA). SMT ((C_2_H_6_N_2_S)_2_·H_2_SO_4_,MW: 278.4, purity: ≥99%) were obtained from Beyotime Biotechnology(haimen, China).

## *In Vivo* Studies

### Animals

Male babl/c 6-8-weeks-old, weighing 20–25 g, were purchased from the Experimental Animal Center of Chinese Academy of Science (Shanghai, China). Animals were provided with standard chow and water and maintained under a 12 h light-dark cycle. The experimental procedures were approved and carried out according to Shanghai University of Traditional Chinese Medicine guidelines for the use and care of experimental animals.

### Group and Drugs

The treatment was as follows: Control Group was injected with saline intrapertoneal (i.p.) with a dose of 0.1 mL/20 g mouse body weight, once daily for 2 weeks consecutively, and served as control. DOX Group was injected i.p. with doxorubicin with a dose of 5 mg/kg i.p. twice/week for 2 weeks^26^. MA Group was injected daily with madecassoside in a dose of 12 mg/kg i.p for 2 weeks^14^. DOX + MA Group was injected with doxorubicin and madecassoside using the same dose schedule mentioned above. Animals were closely monitored on daily basis, weighed twice a week and sacrificed at the end of experiment (after 2 weeks). Blood was collected, and kidney was removed for further studies.

### Evaluation of Antioxidant Enzymes

Kidney tissue protein was determined by BCA (bicinchoninic acid). Kidney tissue supernatant was used for evaluation of enzymes activity. The superoxide dismutase (SOD) and activity of glutathione peroxidase (GSH-PX) in the kidney content were determined using kits from Nanjing Jiancheng Bioengineering Institute.

### Determination of Renal Functions

Venous blood samples were collected after centrifuged at 3000 rpm for 10 min. As a marker of renal function, serum creatinine and blood urea nitrogen (BUN) were measured using colorimetric assay kits according to the manufacturer’s instructions.

### Histopathological Analysis

Formalin-fixed kidney tissues were, paraffin-embedded, cut at 4.6 μm thickness and stained with hematoxylin and eosin (H&E) stain. Histopathological evaluation of the stained tissue sections were performed by a renal histologist, blinded to the sample groups.

## *In Vitro* Studies

### Cell Culture

HK-2, an immortalized proximal tubular cell line derived from normal adult human kidney was obtained from the American Type Culture Collection(ATCC; Manassas, VA, USA). Cell culture were maintained in Dulbecco’s Modified Eagle Media/F12 medium containing 10% heat-inactivated fetal bovine serum(GIBCO, Invitrogen),1% penicillin-streptomycin, pH 7.4, at 37 °C in a humidified 5% CO2 incubator (RCO-3000T, Thermo scientific revco, Asheville, NC, USA). Cell lines were passaged by trypsinization every 3 to 4 days. Cells were used for experiments from passages 5 to 10.

### MTT Assay

Cells were cultured at a density of 1.0 × 10^4^ cells/well in a 96-well plate and treated with various concentrations of drug for the 24 h. Culture medium was then changed with fresh medium containing 3-(4,5-dimethylthiazol-2-yl)-2,5-diphenyltetrazolium bromide (MTT) (final concentration of 0.5 mg/mL). Cells were incubated for another 4 h. Afterward, supernatant was removed, dimethyl sulfoxide (DMSO) (100 μL) was added to each well, and plates were shaken for 15 min. Absorbance of each well was measured using a microplate reader at the wavelength of 570 nm to determine the optical density (OD) value. The MTT assay was performed three times. Cellular viability was calculated as follows: = ODsample/ODcontrol × 100%.

### Nitric Oxide Analysis

Cells were plated at a density of 10 × 10^4^ cells/ml into 96 well plates and incubated for 24 h. Supernatant (50 μL) from each well was transferred to a new well on another 96-well plate and was tested using a NO detection kit (Beyotime Biotechnology, China). Nitrite concentration was determined by spectrophotometry (560 nm) with a standard curve (0–100 mmol/L) by using the Biotek synergy2 system.

### Tunel Assay

Terminal deoxynucleotidyl transferase dUTP nick end labeling (TUNEL) method, is used for detect apoptotic DNA strand breaks, was carried out with the *In Situ* Cell Apoptosis Detection Kit, AP (Sangon Biotech, Shanghai). The cells were treated with DOX and/or MA for 24 h, then all the cells were stained according to the manufacturer’s instructions. Apoptotic cells stained with Purple-blue analyzed were examined by microscope (Olympus Optical Co., Tokyo, Japan). The number of cells with apoptotic bodies was counted in 5 randomly chosen fields at 200 × magnification and the percentage of apoptosis was calculated as the average count of these 5 fields.

### Flow Cytometry

Quantitatively measure of HK-2 apoptosis was detected by phosphatidylserine exposure on cell membrane with Annexin V. After either unstimulated or stimulated with DOX and subsequently treated with MA was studied after 24 h, cells were trypsinized, washed wice with pre-cooled PBS and incubated with a binding buffer containing Annexin V-fluorescein isothiocyanate (FITC) and propidium iodide (PI) (BD Biosciences). Flow cytometry analysis was performed using FACS Aria II (Becton Dickinson). Apoptotic cells were defined as PI-negative and Annexin V-FITC positive.

### Western Blot Analysis

Protein extract from HK-2 cell and kidney tissues was obtained in RIPA lysis buffer containing 0.1% PMSF. HK-2 cells nuclear and cytoplasmic extracts were prepared using the Nuclear Extract Kit (Active Motif). Same amount of samples were separated by SDS-PAGE gel, transferred and immobilized on a polyvinylidene difluoride membrane. The membrane was blocked with 5% nonfat dry milk in Tris-buffered saline containing 0.05% Tween 20 (TBS-T) for 1 h at room temperature. Appropriate primary antibody was followed by incubation of the membranes in overnight at 4 °C. Horseradish peroxidase-conjugated goat anti-mouse IgG secondary antibody (1:5000) (Santa Cruz Biotechnology) was incubated at room temperature for 1 h at room temperature. After the final wash, the immunoreactive bands were detected on ImageQuant 4000mini (GE healthcare) by enhanced chemiluminescence. Data was obtained from three independent experiments.

### Statistical Analysis

Experiments were carried out in triplicate and statistical analysis was accomplished with SPSS software, using stuents’ t test for comparison between two groups. *P* < 0.05 was considered as a significant difference.

## Additional Information

**How to cite this article**: Su, Z. *et al.* Protective effects of madecassoside against Doxorubicin induced nephrotoxicity in vivo and in vitro. *Sci. Rep.* **5**, 18314; doi: 10.1038/srep18314 (2015).

## Figures and Tables

**Figure 1:**
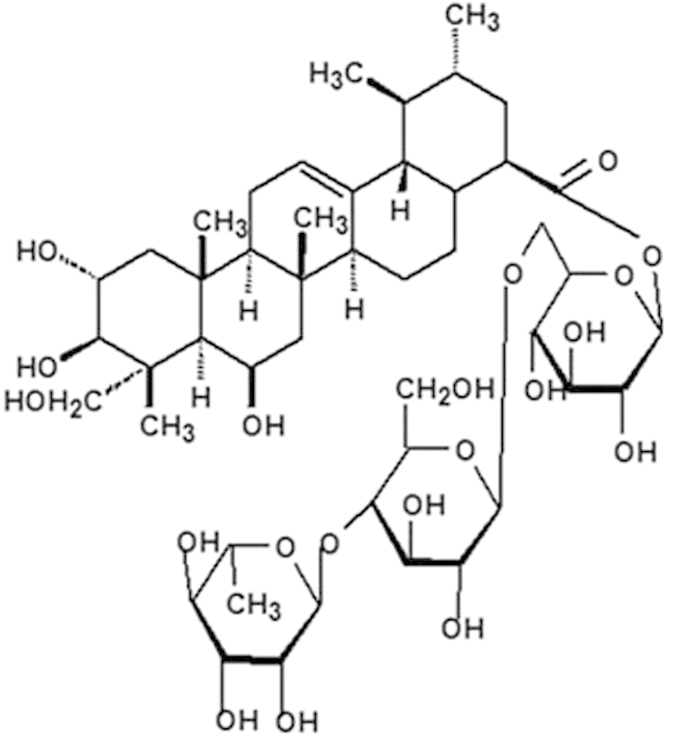
Structure of Madecassoside (MA).
